# Role of Anti-PD-1 Antibodies in Advanced Melanoma: The Era of Immunotherapy

**DOI:** 10.7759/cureus.3700

**Published:** 2018-12-07

**Authors:** Sakshi Sahni, Gautam Valecha, Ankit Sahni

**Affiliations:** 1 Internal Medicine, University of Illinois at Chicago College of Medicine, Chicago, USA; 2 Hematology-Oncology, Staten Island University Hospital, Staten Island, USA; 3 Miscellaneous, Ross University School of Medicine, Knoxville, USA

**Keywords:** pembrolizumab, melanoma, checkpoint inhibitor, nivolumab, immunotherapy

## Abstract

Advanced melanoma is an aggressive skin cancer characterized by poor survival rates and response to cytotoxic chemotherapy. Immune checkpoint inhibitors are novel agents capable of utilizing one’s own immune system to bring about the tumor destruction. Nivolumab and pembrolizumab are fully humanized anti-PD-1 monoclonal antibodies that have shown significant anti-tumor activity in a variety of cancers including melanoma and have significantly improved the survival outcomes in patients with advanced melanoma. In this updated review article, we will discuss the outcomes of various clinical trials evaluating the efficacy and safety of these agents. We will also briefly discuss their mechanism of action and adverse effects.

## Introduction and background

Melanoma is an aggressive skin cancer that has an increased propensity to metastasize resulting in poor survival outcomes. Currently, it is the sixth most common cancer in both men and women in the United States [1]. Although, the incidence of melanoma is rising, most cases are diagnosed at an early stage and are amenable to curative resection. However, the possibility of dissemination significantly increases with stage II, stage III (lymph node involvement) and stage IV disease. Similar to any other malignancy, the survival outcomes worsen with advancing age [2].

The prognosis of melanoma is especially worse with more advanced disease [3]. High dose interleukin-2 (IL-2) was the first agent used in the treatment of metastatic melanoma. It achieved cure in a very small number of patients. However, it was associated with significant toxicity and could only be offered to carefully selected patients at special centers [4]. Moreover, melanoma is highly resistant to cytotoxic chemotherapy. Dacarbazine was approved for treating melanoma in 1976 and was the agent of choice for a long time, given the lack of response to other cytotoxic agents. However, the response rates (RR) were still very poor and the drug never showed any survival advantage when used in metastatic melanoma [5].

The quest for improving the survival outcomes in melanoma led to the discovery of newer pathways involved in the pathogenesis of melanoma that could be potentially targeted by novel agents. One such example includes the mitogen activated protein (MAP) kinase pathway that has been successfully targeted by several newer agents such as the BRAF and MEK inhibitors [6, 7]. However, these drugs can only be used in patients that harbor BRAF mutation and resistance to these agents eventually develops [8]. The goal of immunotherapy, on the other hand, is to unleash one’s own immune system to bring about the tumor destruction. The last decade has witnessed the development of several immunotherapeutic agents for treating melanoma. The development of these agents, especially the checkpoint inhibitors, has drastically improved the survival outcomes in advanced melanoma [9]. Additionally, these drugs have also demonstrated a role in the treatment of other malignancies such as non-small cell lung cancer (NSCLC), urothelial carcinoma, renal cell carcinoma (RCC), colorectal cancers and Merkel cell carcinoma, to name a few [10]. The cytotoxic T-lymphocyte associated protein-4 (CTLA-4) inhibitor ipilimumab and the programmed cell death-1 (PD-1) inhibitors including nivolumab and pembrolizumab are the monoclonal antibodies (MoAbs) that have particularly shown promising results in several clinical trials conducted in patients with advanced melanoma [10].

Ipilimumab was the first checkpoint inhibitor to be tested in phase III trials and showed significant survival advantage when combined with dacarbazine compared to dacarbazine plus placebo [11]. However, it is associated with severe toxicity related to its autoimmune effects. On the other hand, the anti-PD-1 antibodies have demonstrated similar or even better efficacy and offer the advantage of better tolerability compared to ipilimumab, causing ipilimumab to gradually fall out of favor. In this updated review, we will discuss the outcomes of several clinical trials evaluating the efficacy and safety of anti-PD-1 antibodies in melanoma. Additionally, we will also discuss their mechanism of action and unique adverse events (AEs).

## Review

Mechanism of action

The destruction of tumor cells while preventing collateral damage to the host cells is accomplished by a delicate balance between the stimulatory pathways and checkpoints of the immune system. The goal of immunotherapy is to essentially target these checkpoints, inhibit tumor tolerance and bring about the resultant destruction of tumor cells.

The process of tumor destruction begins upon recognition of tumor cells by the antigen presenting cells (APCs). APCs then internalize and process the tumor antigen into major histocompatibility complex (MHC) molecules. The APCs then migrate to lymph nodes, where a complex interaction occurs between the APCs and T cells that ultimately results in the activation and release of T cells into circulation [12]. Once the activated T cell recognizes the tumor antigen associated with the MHC in periphery, it releases cytolytic enzymes and cytokines. This results in proliferation of T cells and recruitment of other cells of immune system ultimately resulting in the tumor destruction.

As mentioned previously, several checkpoints/inhibitory pathways exist in the immune system that prevent the immune cells from destroying the normal host cells (autoimmunity). One such pathway involves interaction between programmed death-1 (PD-1) and programmed death ligand-1 (PD-L1). PD-1 is expressed on the surface of T cells, B cells and natural killer (NK) cells. They are also expressed on the surface of regulatory T cells (Treg) and may enhance their proliferation and function. On the other hand, PD-L1 is expressed on the surface of various cell types including endothelial cells, epithelial cells, cancer cells and hematopoietic cells. PD-1 is upregulated on the surface of T cells upon antigen recognition, whereas PD-L1 expression is upregulated by cytokines and oncogenes. Antigen recognition by T-cell receptor also results in engagement of PD-1 by PD-L1 which activates the signaling pathways that ultimately inhibit tumor apoptosis, T-cell proliferation and function. Additionally, the effector cells are converted to Treg [13]. PD-L1 is found in various cancer cell types including melanoma, lung cancer, hepatocellular carcinoma (HCC), RCC and Merkel cell carcinoma. Checkpoint inhibitors were developed after the discovery of this pathway. Nivolumab and pembrolizumab are anti-PD-1 antibodies, and atezolizumab and durvalumab are anti-PD-L1 antibodies that are capable of inhibiting the interaction between PD-1 and PD-L1, thereby inhibiting this pathway and enhancing immune-mediated tumor destruction.

Nivolumab

Nivolumab is a fully human IgG4 monoclonal antibody that binds to PD-1 receptor and inhibits its activity by blocking its interaction with PD-L1 and PD-L2. As the inhibitory pathway is blocked, T-cell function is enhanced with resultant facilitation of anti-tumor response. Nivolumab has demonstrated activity in various clinical trials conducted in patients with advanced melanoma. It has also been tested in the adjuvant setting as well as in combination with an anti-CTLA-4 antibody, ipilimumab.

Early Phase Studies

The phase I trial conducted in 296 patients afflicted with various cancers such as NSCLC, RCC and melanoma demonstrated an objective response rate (ORR) of 28% (26 of 94 patients) with grade 3 or 4 drug-related adverse events (AEs) in 14% of all the patients [14]. The dose-escalation, cohort expansion phase of this study showed an ORR of 31% with median overall survival (OS) of 16.8 months. One and two-year OS rates were 62 and 43%, respectively. Median duration of response (DOR) was two years and some patients continued to respond even after treatment discontinuation. Apart from durable response, the drug was well tolerated [15]. Another phase 1/2 trial showed that nivolumab given alone or in combination with a peptide vaccine produced an ORR of 26% with a median OS of 18 months in patients with metastatic melanoma who had disease progression on ipilimumab, an anti-CTLA-4 agent [16].

Based on the promising results of the abovementioned trials, nivolumab was further tested in phase III trials to evaluate its efficacy in advanced melanoma both as a frontline agent as well as in those patients previously treated with ipilimumab. It was also tested in combination with ipilimumab.

Previously Untreated Patients

CheckMate 066, a phase III double-blind, randomized controlled trial tested the efficacy of nivolumab in previously untreated patients with advanced melanoma [9]. The investigators had randomly assigned 418 patients to either receive IV nivolumab 3 mg/kg Q2wk plus dacarbazine-matched placebo Q3wk or IV dacarbazine 1000 mg/m^2^ Q3wk plus nivolumab-matched placebo Q2wk. The ORR was 40% in the nivolumab group versus 13.9% in the dacarbazine group. Superior objective response compared to dacarbazine was seen in both PD-L1 positive and PD-L1 negative groups, although the response was better in the PD-L1 positive group. The OS rate at one year was 72.9% in the nivolumab group and 42.1% in the dacarbazine group (HR: 0.42; 99.79% CI: 0.25 to 0.73; P < 0.001). The median progression-free survival (PFS) was also superior in the nivolumab group (5.1 versus 2.2 months, HR: 0.43; 95% CI: 0.34 to 0.56; P < 0.001). Overall, nivolumab was well tolerated by a majority of the patients and its AEs were similar to those seen in the early phase trials. The incidence of drug-related AEs was similar in both the groups (74.3% vs 75.6%). However, the incidence of grade 3 or 4 AEs was significantly lower in the nivolumab group (11.7% vs 17.6%). Health-related quality of life (HRQoL) analysis showed that nivolumab provides long-term quality of survival benefit, compared to dacarbazine in patients with advanced melanoma [17].

Refractory Disease

In the multicenter, randomized controlled, open label, phase III trial conducted by Weber et al. (CheckMate 037), patients with unresectable or metastatic melanoma that had disease progression on ipilimumab (or ipilimumab plus a BRAF inhibitor if they had BRAF V600E mutation) were recruited at 90 sites in 14 different countries [18]. Between December 2012 and January 2014, 631 patients were screened. A total of 405 patients were randomized in a 2:1 ratio to receive either IV nivolumab 3 mg/kg Q2wk (272 patients) or investigator’s choice of chemotherapy (dacarbazine 1000 mg/m^2^ or carboplatin area under curve [AUC] 6 plus paclitaxel 175 mg/m^2^ Q6wk) (133 patients). PD-L1 positivity was defined at 5%. ORR was 27% in nivolumab group and 10% in chemotherapy group. Median DOR was significantly longer in nivolumab group (32 vs 13 months). Efficacy outcomes were better in patients with PD-L1 > 5%. However, there was no significant difference in OS (16 vs 14 months, HR 0.95) or PFS (3.1 vs 3.7 months, HR 1) between the two groups. Grade 3 or 4 AEs were seen in 5% of nivolumab treated patients and 9% of chemotherapy patients.

Combination with Ipilimumab

Phase III trials had indicated that ipilimumab improved OS in patients with advanced melanoma [19]. As mentioned previously, nivolumab was also found to produce durable objective response in patients with advanced melanoma in early phase trials [14]. Preclinical studies had indicated that combined blockade of both PD-1 and CTLA-4 resulted in more potent anti-tumor activity than blockade of either pathway alone [20]. Based on these observations, Wolchok et al. conducted a phase I study to evaluate the safety and efficacy of combination of nivolumab and ipilimumab in 86 patients with advanced melanoma [21]. The drugs were administered both concurrently (53 patients) as well as sequentially (33 patients). The ORR for all 86 patients was 40%. Evidence of any clinical activity including stable disease was seen in 65% of the patients. Grade 3 or 4 treatment-related AEs occurred in 53% of patients in the concurrent-regimen group and 18% of the patients with sequential regimen group. Most of these AEs were reversible and manageable.

Based on the promising results of the abovementioned study, the phase III CheckMate 067 trial was conducted in 945 treatment naive patients with advanced melanoma in the United States and Europe [22]. Patients were randomly assigned to receive one of the following regimens: 3 mg/kg of IV nivolumab Q2wk; 1 mg/kg of IV nivolumab Q3wk plus 3 mg/kg of IV ipilimumab Q3wk for four doses followed by 3 mg/kg of IV nivolumab Q2wk starting cycle three and beyond; or 3 mg/kg of IV ipilimumab Q3wk for four doses. Median PFS was 11.5 months (95% CI: 8.9 to 16.7) in the nivolumab-plus-ipilimumab group compared to 6.9 months (95% CI: 4.3 to 9.5) in the nivolumab group, and 2.9 months (95% CI: 2.8 to 3.4) in the ipilimumab group. At 36 months, the ORR in ipilimumab-nivolumab group was 58% compared to 44% in nivolumab group and 19% in ipilimumab group [23]. The corresponding three-year OS rate was 58% vs 52% vs 34% in the three groups. The median OS was not reached in the combination group and was 37.6 months in the nivolumab group and was 19.9 months in the ipilimumab group. At three years, the median DOR was not reached in any of the nivolumab containing groups and was 19.3 months in ipilimumab group.

Adjuvant Setting

The promising efficacy of immunotherapy in advanced melanoma led to the evaluation of checkpoint inhibitors in the adjuvant setting. In the EORTC 18071 placebo-controlled trial, ipilimumab significantly improved the recurrence-free survival (RFS), five-year distant metastasis-free survival as well as OS compared to placebo in patients with resected high-risk melanoma [24, 25]. Although, no trials were conducted to compare ipilimumab with interleukin-2 (IL-2), the magnitude of OS benefit seen with ipilimumab led to its approval for the adjuvant treatment of stage III melanoma. However, the toxicity associated with it was significant. Based on the promising results and favorable safety profile seen with nivolumab in advanced melanoma, CheckMate 238 trial was conducted to compare its effectiveness with ipilimumab in patients with stage IIIB, IIIC or IV resected melanoma [26]. The 12-month RFS was 70.5% in the nivolumab group and 60.8% in the ipilimumab group (HR for disease recurrence or death, 0.65; 97.56% CI: 0.51 to 0.83; P < 0.001). The updated results presented at American Society of Clinical Oncology (ASCO) also showed a superior 24-month RFS rate in nivolumab group compared to ipilimumab group (63% versus 50%, HR: 0.66, 95% CI: 0.54-0.81). (Oral abstract: Weber JS, Mandalà M, Del Vecchio M, et al. Adjuvant Therapy with Nivolumab (NIVO) versus Ipilimumab (IPI) after Complete Resection of Stage III/IV Melanoma: Updated Results from a Phase III Trial (CheckMate 238). American Society of Clinical Oncology; June 04, 2018). Besides the superior efficacy, the rate of treatment-related grade 3 or 4 AEs was 14% in nivolumab group and 46% in ipilimumab group. Treatment-related AEs led to treatment discontinuation in 4% of the patients in nivolumab group and 30% of the patients in ipilimumab group.

Based on these promising results, the US Food and Drug Administration (FDA) in December 2017, granted regular approval to nivolumab for the adjuvant treatment of patients affected with melanoma with lymph node involvement or in patients with metastatic disease who have undergone definitive resection of all the disease sites. Initially, a dose of 240 mg of IV nivolumab given every two weeks was approved. Subsequently, an alternative schedule of 480 mg given every four weeks was also approved.

Pembrolizumab

Pembrolizumab is also a fully humanized anti-PD-1 monoclonal antibody that selectively binds to PD-1 receptor on T cells, preventing it from binding PD-L1 on tumor cells. This prevents tumor tolerance and aids in the destruction of cancer cells.

Early Phase Studies

Pembrolizumab has shown efficacy in several cancers including NSCLC, Merkel cell carcinoma, urothelial carcinoma, head and neck cancer, microsatellite instability-high colorectal cancer, cervical carcinoma and melanoma. The multicohort phase IB KEYNOTE-001 study conducted in 655 patients with advanced or metastatic melanoma (both ipilimumab naive and ipilimumab treated) showed an ORR of 33%, 12-month PFS rate of 35%, and median OS of 23 months. Grade 3 or 4 treatment-related AEs occurred in 14% of the patients [27]. A subsequent study examining the relationship between the activity of pembrolizumab and PD-L1 expression in the patients enrolled in KEYNOTE-001 trial showed that PD-L1 expression in pretreatment tumor biopsy samples correlated with response rate, PFS, and OS. However, patients with PD-L1 negative tumors also had durable response [28].

Refractory Disease

In the phase II randomized controlled KEYNOTE-002 trial, 540 ipilimumab refractory patients (or after progressing on targeted therapy if BRAF mutation present) with advanced melanoma were randomly assigned to receive pembrolizumab 2 mg/kg, pembrolizumab 10 mg/kg, or investigator-choice chemotherapy [29]. PFS was significantly improved in both the pembrolizumab groups compared to the chemotherapy group. The six-month PFS was 34% in the pembrolizumab 2 mg/kg group, 38% in the 10 mg/kg group, and 16% in the chemotherapy group. At 28 months of follow-up, 55% of the patients had crossed over to pembrolizumab group [30]. The ORR were 22%, 26%, and 4%, respectively, for pembrolizumab 2 mg/kg, pembrolizumab 10 mg/kg, and chemotherapy. The 24-month PFS rates for these three groups were 16%, 22%, and 0.6%, respectively. There was a non-statistically significant improvement in OS in pembrolizumab groups compared to the chemotherapy group. Median OS in pembrolizumab 2 mg/kg, pembrolizumab 10 mg/kg and chemotherapy group was 13.4, 14.7 and 11.0 months, respectively while the two-year OS rates were 36%, 38% and 30%. Pembrolizumab was better tolerated than chemotherapy. Grade 3 or 4 treatment-related AEs occurred in 13.5%, 16.8% and 26.3% of the patients in pembrolizumab 2 mg/kg, pembrolizumab 10 mg/kg and chemotherapy group, respectively.

Previously Untreated Patients

Pembrolizumab was subsequently tested in patients with ipilimumab naive advanced melanoma. In the phase III KEYNOTE-006 trial, 834 patients were randomly assigned in a 1:1:1 ratio to receive 10 mg/kg IV pembrolizumab every two weeks or 10 mg/kg IV pembrolizumab every three weeks or four doses of 3 mg/kg IV ipilimumab every three weeks [31]. Treatment with pembrolizumab was continued for two years as long as there was no disease progression or severe toxicity. Approximately, 35% of the patients had BRAF V600 mutation and half of these had received targeted therapy previously. Patients were eligible to participate only if they did not receive more than one systemic therapy. Patients previously treated with ipilimumab or anti-PD-1/PD-L1 agents were excluded from the study. At 23 months, pembrolizumab was associated with better OS than ipilimumab [32]. No significant differences in efficacy outcomes were seen between the two pembrolizumab groups. The 24-month PFS rate was 31% in the Q2wk pembrolizumab group, 28% in the Q3wk pembrolizumab group, and 14% in ipilimumab group (HR for pembrolizumab versus ipilimumab 0.61, 95% CI: 0.50-0.75). The four-year survival data was presented at the 2018 ASCO annual meeting (Oral abstract: Long GV, Schachter J, Ribas A, et al. 4-year Survival and Outcomes after Cessation of Pembrolizumab (pembro) after 2 Years in Patients (pts) with Ipilimumab (ipi)-Naive Advanced Melanoma in KEYNOTE-006. American Society of Clinical Oncology; June 04, 2018). OS was significantly prolonged with pembrolizumab compared to ipilimumab. The four-year OS rate was 42% in pooled pembrolizumab groups and 34% in ipilimumab group. The ORR was also significantly better in pembrolizumab group (42 vs 17%). Overall, only 103 of the 556 patients (19%) could complete the two years course of pembrolizumab, of which 89 (86%) had no progression at a median follow-up of 20 months. Pembrolizumab was also better tolerated than ipilimumab. The rate of grade 3-5 treatment-related AEs was lower in the pembrolizumab groups (13.3% and 10.1%) than in the ipilimumab group (19.9%) [31]. Furthermore, HRQoL was better maintained in patients on pembrolizumab compared to those who received ipilimumab [33].

Combination with Ipilimumab

After the improved efficacy of nivolumab and ipilimumab combination was demonstrated, the combined efficacy and toxicity of ipilimumab plus pembrolizumab was tested in a phase IB trial in patients from 12 centers in the US, Australia and New Zealand [34]. Patients received IV pembrolizumab 2 mg/kg plus IV ipilimumab 1 mg/kg Q3wk for four doses, followed by IV pembrolizumab 2 mg/kg Q3wk for up to two years or disease progression or intolerable toxicity. The ORR was 61% at median follow-up of 17 months. The one-year PFS rate was 69% and OS rate was 89%. Immune-related adverse events (IrAEs) of any grade occurred in 60% of the patients, and of grade 3-4 occurred in 27% of the patients.

Adjuvant Setting

Similar to nivolumab, pembrolizumab was also tested as an adjuvant agent. In the KEYNOTE-054 trial, 1019 patients with resected stage III melanoma were randomly assigned to receive either 200 mg of IV pembrolizumab every three weeks for 18 doses (approximately for one year) or a matched placebo [35]. At a median follow-up of 15 months, pembrolizumab was associated with longer RFS. The one-year RFS rate was 75.4% in pembrolizumab group versus 61% in placebo group. Subgroup analysis showed that the PD-L1 expression did not correlate with anti-PD-1 activity. Pembrolizumab improved the RFS in both PD-L1 positive and PD-L1 negative subgroups. Grade ≥ 3 AEs were more common with pembrolizumab than with placebo (14.7% vs 3.4%).

Pembrolizumab is now being compared with high-dose interferon (IFN) or high-dose ipilimumab in patients with completely resected high-risk stage III or IVA disease (NCT02506153).

Adverse effects

Treatment with checkpoint inhibitors can result in a unique group of adverse effects related to autoimmune destruction of various tissues. These irAEs can involve any organ system. Dermatologic toxicity characterized by various types of skin rashes is the commonest irAEs. Other irAEs include colitis, pneumonitis, hepatotoxicity and endocrinopathies involving thyroid, hypothalamus/pituitary and adrenal glands. Rarely, other organ systems such as kidneys, pancreas, central and peripheral nervous system, heart, eyes and musculoskeletal system can be involved. Generally, the incidence of these AEs is lower with anti-PD-1/PD-L1 agents compared to ipilimumab [36]. The incidence is also higher when these two classes of drugs are used in combination [23]. The severity of these AEs is graded according to Common Terminology Criteria for Adverse Events (CTCAE). The ASCO organized a multidisciplinary, multiorganizational panel of experts that conducted an extensive review of the literature and proposed both general and organ-system specific recommendations for the management of AEs seen with checkpoint inhibitors [37]. As per their guidelines, in general, the moAB may be continued with close observation in cases of grade 1 AEs with the exception of certain neurologic, cardiac and hematologic AEs. Checkpoint inhibitors should be held in most cases of grade 2 toxicities and may be resumed once the toxicity reverts to grade 1 or resolves. Steroids may be used. Immune checkpoint inhibitors (ICPIs) should be held in cases of grade 3 toxicity. High dose steroids are the mainstay of treatment. Other immunomodulators like infliximab may be used in refractory cases. The ICPIs should be permanently discontinued in patients' grade 4 toxicity except in cases of endocrinopathies that are well controlled with hormone replacement. Non-immune systemic adverse effects include fatigue and infusion reaction. Fatigue is the commonest adverse effect of anti-PD-1/PD-L1 antibodies [38]. It is more commonly seen with ipilimumab compared to anti-PD-1 antibodies. The incidence is also higher when these two classes of drugs are combined with each or with other drugs. When patients present with fatigue, it is important to rule out other conditions like hypothyroidism or primary adrenal insufficiency. It is also important to rule out the immune-mediated endocrinopathies resulting from ICPIs. Mild infusion reactions including fever, chills and headache have been reported with the use of anti-PD-1 inhibitors. However, the incidence of severe life-threatening reaction is extremely rare. Acetaminophen or other nonsteroidal anti-inflammatory drugs (NSAIDs) may be used for mild reactions. Intravenous antihistamines or steroids may be used for severe reactions.

## Conclusions

For a long time, advanced melanoma was associated with extremely poor prognosis characterized by poor survival and disappointing response to conventional chemotherapy. Over the last 10 years, the concept of harnessing one’s own immune system to kill the tumor cells has translated into the identification of checkpoints in the immune pathway and development of novel agents that can target these checkpoints. These agents have drastically improved the survival outcomes in patients with advanced melanoma. Ipilimumab was the first ICPI to be approved for use in advanced melanoma. However, the newer anti-PD-1 agents like nivolumab and pembrolizumab because of superior efficacy and safety profile are gradually replacing ipilimumab in metastatic as well as adjuvant setting. However, ipilimumab may still have a role when used in combination with the anti-PD-1 agents. Current trials are now evaluating the role of these drugs in combination with other agents like the targeted therapy [NCT02910700] as well as intralesional therapy with the oncolytic virus talimogene laherparepvec (T-VEC) [NCT02263508]. Although, the ICPIs are better tolerated than cytotoxic chemotherapy, they are associated with unique adverse effects related to the immune activation and are capable of affecting any organ system. In our opinion, the next 10 years will witness their increasing role in melanoma in a variety of settings, and in combination with other modalities. At the same time, we will need more studies to identify the patient population that will have a better response to these agents. Finally, the future studies should also focus on identifying other pathogenetic and immune pathways with the intent to develop more efficacious agents.
